# The Phenotypic Landscape of *Phloeosinus baumanni*: Spatial Patterns of Morphological Variation and Their Implications for Ecology and Taxonomy

**DOI:** 10.3390/insects17060625

**Published:** 2026-06-14

**Authors:** Montserrat Cervantes-Espinoza, Edwin R. Ariza-Marín, Tonatiuh Santos-Neria, Mauricio Pérez-Silva, Rodolfo J. Cancino-López, Osiris Valerio-Mendoza, Alba R. Dueñas-Cedillo, Enrico Alejandro Ruiz, Luis Gerardo Cuellar-Rodríguez, Israel Yerena-Yamallel, Francisco Armendáriz-Toledano

**Affiliations:** 1 Laboratorio de Ecología, Departamento de Zoología, Escuela Nacional de Ciencias Biológicas, Instituto Politécnico Nacional, Mexico City 11340, Mexico; montbio20@gmail.com (M.C.-E.); eruizc@ipn.mx (E.A.R.); 2 Colección Nacional de Insectos, Departamento de Zoología, Instituto de Biología, Universidad Nacional Autónoma de México, Mexico City 04510, Mexico; erarizam@gmail.com (E.R.A.-M.); santosneria@gmail.com (T.S.-N.); mau_2986@hotmail.com (M.P.-S.); osirisvaleriom@gmail.com (O.V.-M.); 3 Facultad de Estudios Superiores Iztacala, Universidad Nacional Autónoma de México, Mexico City 54090, Mexico; 4 Facultad de Ciencias Forestales, Universidad Autónoma de Nuevo León, Linares 67700, Mexico; cancinorodolfoj@gmail.com (R.J.C.-L.); albaduenascedillo@gmail.com (A.R.D.-C.); entomolab@gmail.com (L.G.C.-R.)

**Keywords:** phenotypic variation, sexual dimorphism, bark beetles, intraspecific differences, Mexican cypress

## Abstract

Morphological characters are widely used for species delimitation in Scolytinae, yet their stability under intraspecific variation remains poorly understood. Here, we analyzed phenotypic variation in specimens currently assigned to *Phloeosinus baumanni* across its distribution in Mexico to evaluate the effects of sexual dimorphism and locality on morphological traits. A total of 25 characters were examined using univariate and multivariate analyses. Pronounced sexual dimorphism was detected, with significant differences in more than half of the evaluated traits and a clear separation between sexes in multivariate space, particularly in body size, cephalic proportions, and elytral declivity features. Locality-associated variation was also detected, particularly within the better-sampled localities of the Transmexican Volcanic Belt. However, localities showed broad overlap in morphospace, with no consistent clustering across biogeographic provinces. Several traits commonly used for species delimitation exhibited substantial variation associated with both sex and geography. These results indicate sex- and locality-associated phenotypic variation among specimens currently assigned to *P. baumanni* and highlight the risk of taxonomic overestimation when variable morphological traits are overemphasized. Incorporating intraspecific variability into taxonomic frameworks is essential to improve species delimitation in Scolytinae.

## Simple Summary

This study examines a bark beetle associated with cypress trees and widely distributed across Mexico and Central America. Species in this group are often identified using external morphological traits, but these traits may vary within species and lead to uncertain classifications when interpreted without geographic or sexual context. We evaluated variation in body size and external morphology in specimens currently assigned to *Phloeosinus baumanni* to determine whether these differences were associated with sex or locality. We found clear differences between males and females, as well as morphological variation among localities. However, these differences overlapped in morphospace and did not form clearly separate morphological groups. These results suggest that natural variation within species should be considered when using morphology for identification. Additional molecular evidence will be necessary to fully test species boundaries. Accurate species identification is important for forest management, pest control, and biodiversity conservation in ecosystems where bark beetles play important ecological roles.

## 1. Introduction

Bark beetles of the subfamily Scolytinae (Coleoptera: Curculionidae) constitute one of the most diverse groups of insects associated with forest ecosystems, with 6516 species described worldwide [1,2,3,4]. North and Central America harbour over 1400 species, highlighting Mexico as one of the countries with high diversity, with 962 species recorded [5,6,7,8]. Despite this species richness, inter- and intraspecific morphological variation remains insufficiently evaluated, particularly in taxa of forestry relevance such as *Phloeosinus* Chapuis, 1869 [9]. This genus is distributed in the Holarctic region and in some areas of the Pacific. It includes 80 described species, but 66 are taxonomically valid [1,2,3,10,11,12], and 9 of them are registered for Mexico [6,13,14], some of which are associated with ecological and sanitary impacts on plantations in cypress and cedar forests due to their ability to colonize living or weakened trees [12].

Among these species, *Phloeosinus baumanni* Hopkins, 1905, stands out for its wide geographic distribution and for being categorized as a species of forestry importance, under Mexican regulations [15]. The distribution of this species extends from southeastern Arizona in the United States to Central America, with confirmed records in various states in the central and southern regions of Mexico [8,13]. Identification of this species has been based on external characteristics, primarily by the presence of tubercles on the elytral declivity, which are conspicuous cuticular protuberances, as well as on the host plant identity, the Mexican cypress *Hesperocyparis lusitanica* (Mill.) Bartel, 2009 [1,10,12,16].

*Phloeosinus baumanni* displays a remarkable external morphological resemblance to other species, such as *Phloeosinus cupressi* Hopkins, 1903, and *Phloeosinus variolatus* Bruck, 1931, which share similar elytral sculpture patterns and host associations [1,10,16]. This similarity is taxonomically relevant because the separation of these species has traditionally relied on subtle external characters, including the development and distribution of crenulations and tubercles on the elytral disc and declivity, differences in vestiture density, and other external traits that may vary among individuals. In this context, *P. variolatus* represents an important comparative reference because it illustrates how closely related or morphologically similar species within *Phloeosinus* may be distinguished by combinations of small diagnostic characters rather than by a single conspicuous trait. Therefore, evaluating the range of variation in *P. baumanni* is necessary to determine whether the characters currently used to recognize this species remain stable across sex and geography, or whether some of them overlap with the variation observed in morphologically similar species. Phenotypic diversity within *P. baumanni* has been scarcely explored since its original description and rarely examined explicitly, across its geographical distribution and sexual dimorphism [10,16].

A recent study within the genus showed that populations of *Phloeosinus serratus* (LeConte, 1868) exhibit pronounced phenotypic differentiation associated with locality and host, forming discrete morphological groups when analyzed using linear measurements, qualitative traits, and shape data [17]. These differences involve characters traditionally used in species delimitation, including elytral sculpture, vestiture patterns, and body measurements [1]. This example highlights that morphological variation in *Phloeosinus* may be substantial within named species and should be interpreted carefully when used to infer taxonomic boundaries. This pattern underscores the need to re-evaluate the interpretation of morphological variability within the genus, particularly when assessed without an explicit geographic context.

The study of morphological variability at the intraspecific level is essential for evaluating the breadth and consistency of characters used in species recognition, especially in groups where sexual dimorphism and environmentally influenced variation may affect trait expression [18,19]. A detailed assessment of this variability helps distinguish sex-related differences, locality-associated variation, and potential morphological discontinuities, thereby avoiding inaccurate interpretations of species boundaries [20,21,22,23]. In this context, incorporating a geographic perspective allows the identification of morphological characters that remain stable across geographic and sexual variation, as well as those that are highly labile and potentially influenced by environmental conditions or sex-specific differentiation [22,23]. Documenting these patterns contributes to refining diagnostic criteria at the intrageneric level, reducing the risk of overestimating species diversity based on highly variable traits, and improving the reliability of morphological identification [20,21,23,24]. Furthermore, understanding spatial patterns of phenotypic variation provides valuable information for ecological, biogeographical, and forest management studies, where accurate species recognition is essential for interpreting patterns and making informed decisions [25,26,27]. Given the broad geographic distribution of *P. baumanni*, two alternative, but non-mutually exclusive, patterns may explain its morphological variability. First, phenotypic variation may be broadly overlapping across biogeographic provinces. Second, variation may be arranged into discrete morphological clusters associated with particular biogeographic units, indicating stronger phenotypic differentiation among sampled areas. Within this morphological framework, we evaluated whether phenotypic variation in specimens assigned to *P. baumanni* shows broad overlap or discrete clustering across biogeographic provinces and among localities within the Transmexican Volcanic Belt. Specifically, we analyzed the structure of phenotypic variation at both coarse and fine spatial scales, among and within biogeographic provinces, respectively, following the biogeographic regionalization proposed by Juan J. Morrone, Tania Escalante, and Gonzalo Halffter [28,29,30,31,32]. We tested whether morphological variation in *P. baumanni* shows broad morphospace overlap or instead displays discrete clusters associated with biogeographic units.

## 2. Materials and Methods

### 2.1. Specimen Selection

To examine morphological variation in *P. baumanni* through its distribution, we analyzed a total of 254 adult specimens (128 females and 126 males) from 21 localities in Mexico and Guatemala (Table 1). Specimens’ identification was corroborated using taxonomic keys [1]. Then, we sexed specimens using diagnostic characters, such as armed first interstriae base of the declivity in males. In contrast, in females, they bear teeth equal in size to those on the third interstriae [17]. Specimens were obtained both from infested host trees in the field and from entomological collections, including the: Colección Nacional de Insectos, Instituto de Biología Universidad Nacional Autónoma de México (CNIN), División de Ciencias Forestales, Universidad Autónoma Chapingo (UACH), Colección Nacional de Referencia de Insectos de Importancia Forestal y Cuarentenaria de la Secretaria del Medio Ambiente (LARSF-SEMARNAT), Colección Entomológica del Instituto Nacional de Investigaciones Forestales Agrícolas y Pecuarias, Coyoacán (INIFAP), and Escuela Nacional de Ciencias Biológicas, del Instituto Politécnico Nacional (ENCB).

Records originally associated with *Cupressus lusitanica* were reassigned to *Hesperocyparis lusitanica*, following current taxonomic and phylogenetic evidence, supporting the placement of New World species within *Hesperocyparis* [33,34].

Because the available material came from both field sampling and museum collections, the number of specimens was uneven among localities and biogeographic provinces. Therefore, ordination patterns involving sparsely sampled regions were interpreted cautiously, and detailed locality-level analyses were restricted to the Transmexican Volcanic Belt localities with at least 14 specimens. This threshold was used to reduce the influence of extremely small samples on locality-level comparisons and to avoid overinterpreting ordination patterns from poorly represented sites.

### 2.2. Geographical Coverage

**Figure 1:**
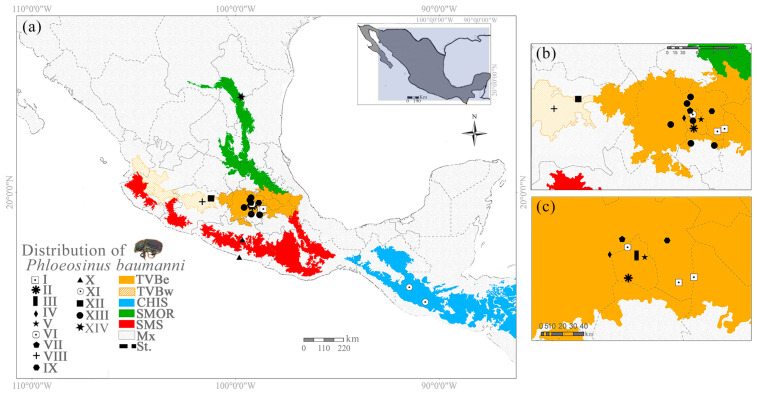
Geographic distribution of *Phloeosinus baumanni* in central and southern Mexico and Guatemala. (**a**) General distribution map of *P. baumanni*. (**b**) Enlarged view of the Transmexican Volcanic Belt showing the detailed placement of locality records. (**c**) Further enlargement of the eastern Transmexican Volcanic Belt, highlighting the precise distribution of records. Abbreviations: I, Amecameca; II, Coyoacán; III, Cuauhtémoc; IV, Huixquilucan; V, Iztapalapa; VI, Miguel Hidalgo; VII, Naucalpan; VIII, Pátzcuaro (Michoacán); IX, Texcoco; X, localities in Guerrero; XI, localities in the Chiapas Highlands; XII, remaining locality in Michoacán; XIII, remaining localities in the eastern Transmexican Volcanic Belt; XIV, locality in Nuevo León; TVBe, Transmexican Volcanic Belt (eastern sector, orange); TVBw, Transmexican Volcanic Belt (western sector, yellow stripes); CHIS, Chiapas Highlands (blue); Mx, Mexico (grey area); SMOR, Sierra Madre Oriental (green); SMS, Sierra Madre del Sur (red); St, division of Mexican states (gray dashed line).

To illustrate the known distribution of *P. baumanni* in America, we generated a distribution map based on the geographic coordinates of each sampling point. Maps were constructed using ArcGIS version 10.8 (Esri, Redlands, CA, USA) [35]. Biogeographic regionalization shapefiles of the Americas were used as base layers, encompassing the Nearctic and Neotropical regions and their corresponding biogeographic provinces, following Escalante et al. [28] and Morrone et al. [29]. To improve visualization and avoid saturation of the maps, only those biogeographic provinces containing sampling records or located near sampling sites were included, maintaining the original nomenclature of the proposed regionalization. Final editing and graphical standardization were carried out in Adobe Illustrator 2021 (Adobe Inc., San Jose, CA, USA) [36]. In the resulting maps, each sampling locality was represented using distinct symbology to differentiate those sites where morphological variation was observed in both females and males (Figure 1).

### 2.3. Morphological Characterization

**Figure 2:**
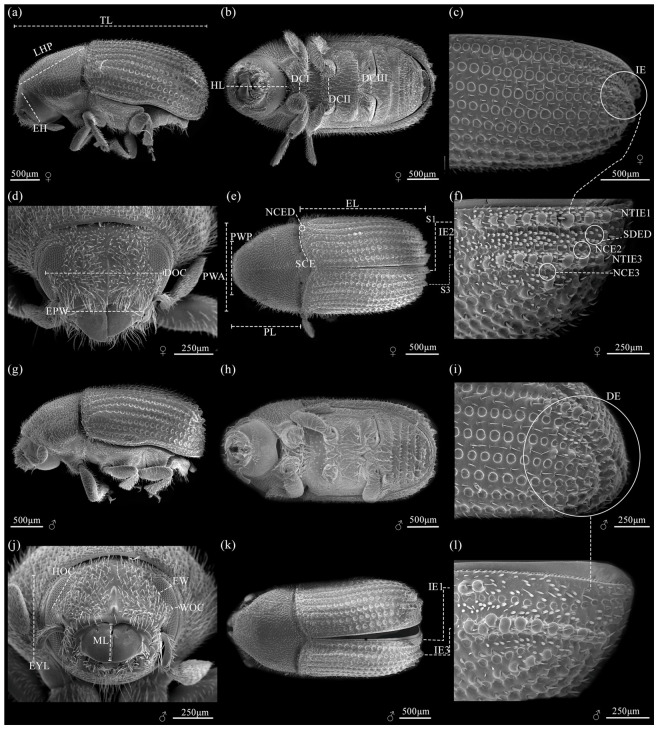
Scanning electron micrographs of *Phloeosinus baumanni* showing morphological features analyzed from female (**a**–**f**) and male (**g**–**l**) specimens. (**a**) Female, lateral habitus. (**b**) Female, ventral habitus. (**c**) Female, elytral disc. (**d**) Female, frontal view of head. (**e**) Female, dorsal habitus. (**f**) Female, elytral declivity. (**g**) Male, lateral habitus. (**h**) Male, ventral habitus. (**i**) Male, elytral disc. (**j**) Male, frontal view of head. (**k**) Male, dorsal habitus. (**l**) Male, elytral declivity. Abbreviations: DCI, distance between procoxae; DCII, distance between mesocoxae; DCIII, distance between metacoxae; DE, dorsal elytral region; DOC, distance between ocular canthus; EH, eye height at lateral side; EL, elytral length; EPW, epistomal width; EW, eye width; EYL, eye length; HL, head length; HOC, height of ocular canthus; IE, elytral interstria region; IE1, first elytral interstria region; IE2 s interstriae; IE3, third elytral interstria region; LHP, head–pronotum length; ML, mandible length; NCED, number of crenulations on the edge of the elytral disc; NCE2, number of crenulations on the second elytral striae; NCE3, number of crenulations on the third elytral striae; NTIE1, number of tubercles on the first elytral interstriae; NTIE3, number of tubercles on the third elytral interstriae; PL, pronotal length; PWA, pronotal width in anterior region; PWP, pronotal width in posterior region; S1-striae; S3 third striae; SCE, shape of crenulations on the elytral disc; S1 first striae; SDED, scale density on the elytral declivity; TL, total body length; WOC, width of ocular canthus.

**Figure 3:**
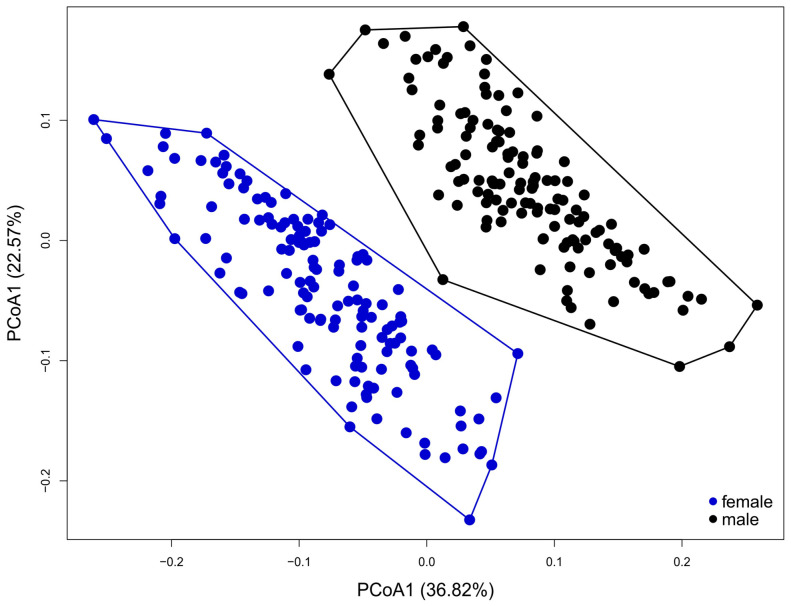
Multivariate ordination analysis for *Phloeosinus baumanni* to illustrate sexual dimorphism. Principal coordinate analysis colouring by sex.

**Figure 4:**
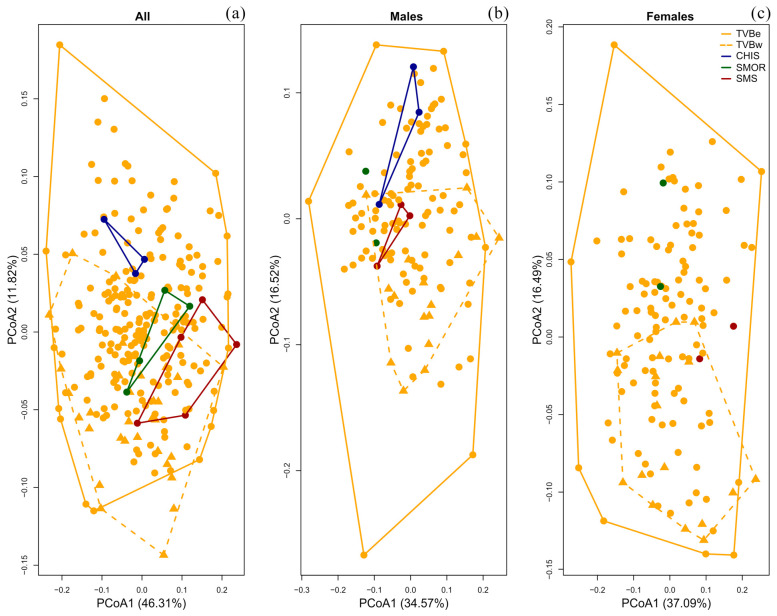
Multivariate ordination analyses for *Phloeosinus baumanni* to illustrate grouping by biogeographic provinces. Principal coordinate analyses colouring by biogeographic province. (**a**) All specimens with variables of non-sexual traits. (**b**) Males with sexually dimorphic traits. (**c**) Females with sexually dimorphic variables. Abbreviations: CHIS, Chiapas Highlands (blue); SMOR, Sierra Madre Oriental (green); SMS, Sierra Madre del Sur (red); TVBe, Transmexican Volcanic Belt (eastern sector, solid line and circles in orange); TVBw, Transmexican Volcanic Belt (western sector, dashed line and triangles in orange).

**Figure 5:**
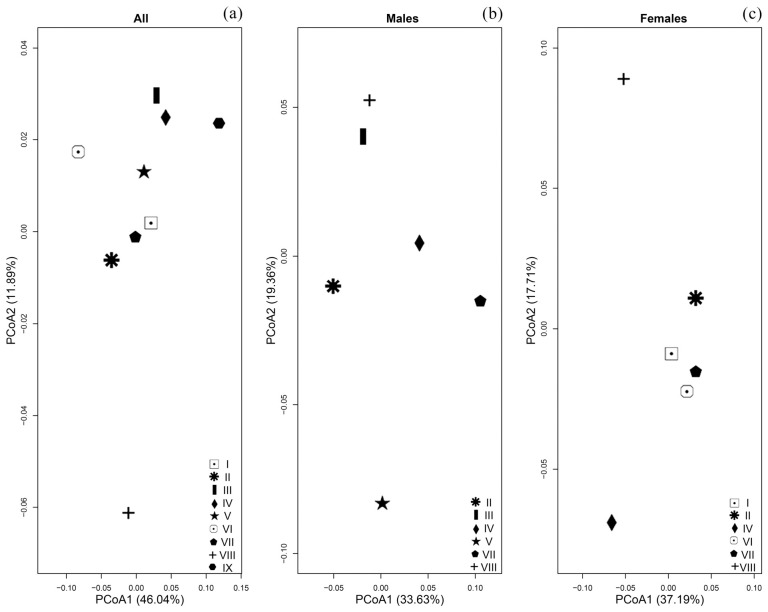
Multivariate ordination analyses for *Phloeosinus baumanni* to illustrate group means (populations) within the Transmexican Volcanic Belt. Principal coordinate analyses. (**a**) All specimens with variables of non-sexual traits. (**b**) Males with sexually dimorphic traits. (**c**) Females with sexually dimorphic variables. Abbreviations: I, Amecameca; II, Coyoacán; III, Cuauhtémoc; IV, Huixquilucan; V, Iztapalapa; VI, Miguel Hidalgo; VII, Naucalpan; VIII, Pátzcuaro (Michoacán); IX, Texcoco.

**Figure 6:**
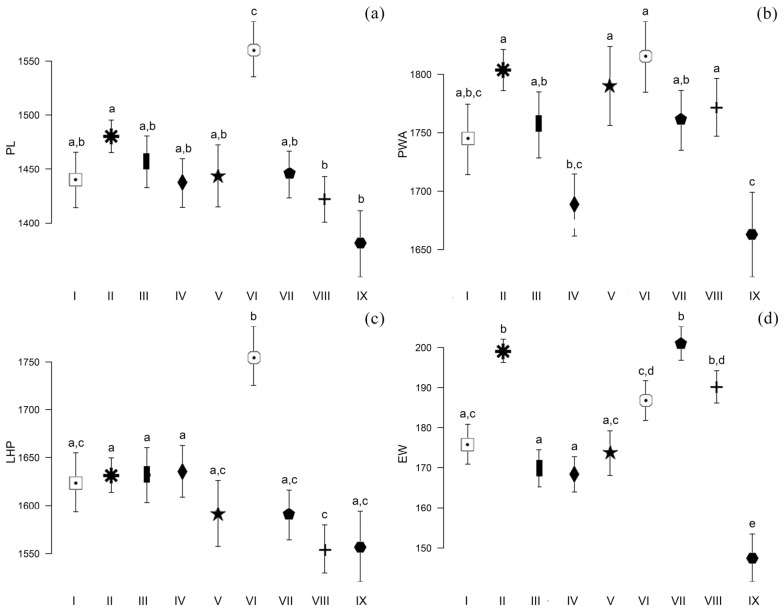
Univariate differences among populations for non-sexually dimorphic traits. Error bars represent the mean and standard error per population. (**a**) Pronotal length (PL). (**b**) Pronotal width in anterior region (PWA). (**c**) Length of head-pronotum (LHP). (**d**) Eye width (EW). Abbreviations: I, Amecameca; II, Coyoacán; III, Cuauhtémoc; IV, Huixquilucan; V, Iztapalapa; VI, Miguel Hidalgo; VII, Naucalpan; VIII, Pátzcuaro (Michoacán); IX, Texcoco. Different letters above the error bars indicate statistically significant differences among localities. For sexually dimorphic traits in males, the variables were head length (HL: df = 5, F-value = 6.468, *p*-value < 0.001; Table S2), distance between ocular canthus (DOC: df = 5, F-value = 2.515, *p*-value = 0.035; Table S2), epistomal width (EPW: df = 5, F-value = 4.602, *p*-value < 0.001; Table S2), and number of crenulations on the second elytral striae (NCE2: df = 5, F-value = 43.753, *p*-value < 0.001; Table S2). Specimens from Naucalpan (VII) had the shortest head (Figure 7a), specimens from Iztapalapa (V) had the ocular canthus furthest away (Figure 7b) and more crenulation on the second elytral striae (Figure 7d), and the specimens from Coyoacán (II) and Miguel Hidalgo (VI) had the widest epistome (Figure 7c). The remaining localities formed a homogeneous group (Figure 7).

**Figure 7:**
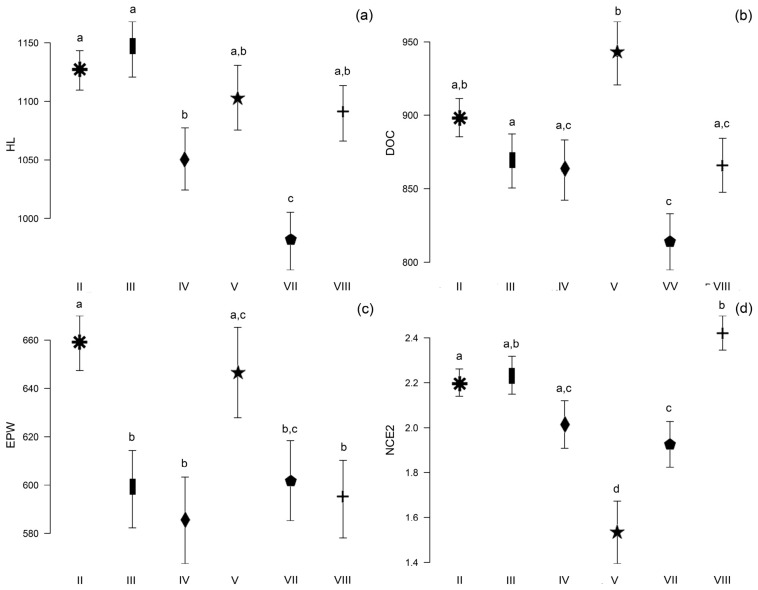
Univariate differences among populations in males for sexually dimorphic traits. Error bars represent the mean and standard error per population. (**a**) Head length (HL). (**b**) Distance between ocular canthus (DOC). (**c**) Epistomal width (EPW). (**d**) Number of crenulations on the second elytral striae (NCE2). Abbreviations: II, Coyoacán; III, Cuauhtémoc; IV, Huixquilucan; V, Iztapalapa; VII, Naucalpan; VIII, Pátzcuaro (Michoacán). Different superscript letters indicate statistically significant differences among localities.

**Figure 8:**
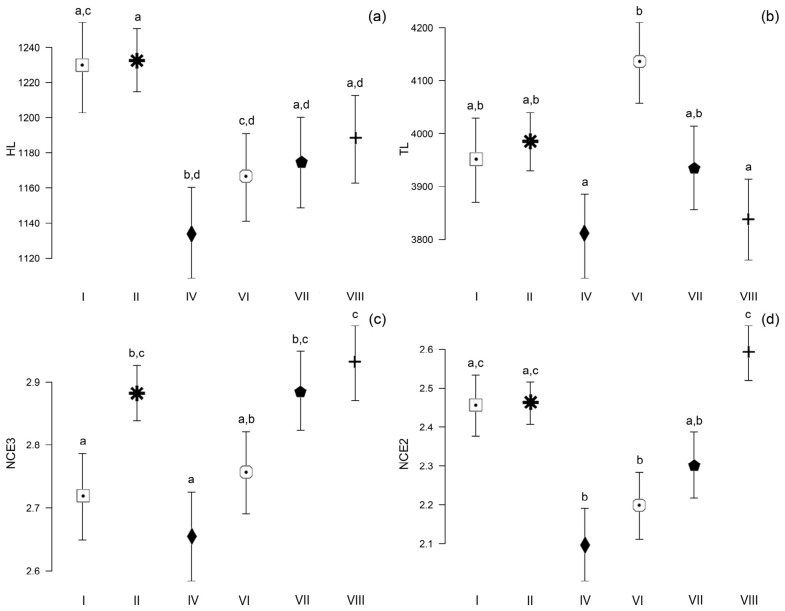
Univariate differences among populations in females for sexually dimorphic traits. Error bars represent the mean and standard error per population. (**a**) Head length (HL). (**b**) Total length (TL). (**c**) Number of crenulations on the third elytral striae (NCE3). (**d**) Number of crenulations on the second elytral striae (NCE2). Abbreviations: I, Amecameca; II, Coyoacán; IV, Huixquilucan; VI, Miguel Hidalgo; VII, Naucalpan; VIII, Pátzcuaro (Michoacán). Different superscript letters indicate statistically significant differences among localities.

To characterize the morphology variation in *P. baumanni*, 25 external morphological characters were selected from the head, pronotum, and elytra. The characters were defined with different methods (measurements, counts, and qualitative descriptions) as follows: 18 were continuous, five were counts, and two were binary characters. Specimens were examined under a stereoscopic microscope Koppace 50 at 40× magnification for counts and binary characters. High-resolution images of dorsal, lateral, and ventral habitus were obtained using a digital Rising Cam 16 MP digital camera (RisingCam, Ningbo, China) attached to a Rising Tech UrCMOS stereoscope, to obtain measurements. To optimize data collection, we recorded the Cartesian coordinates of spatially homologous points located at the beginning and end of each continuous variable, as well as the scale bar, using tpsDig2 [37]. Then, measurements were taken with the software PAST ver. 1.95 [38]. To complement morphological observations, scanning electron micrographs were obtained from five males and five females using a Hitachi S-2469N scanning electron microscope (Hitachi, Tokyo, Japan), allowing detailed visualization of diagnostic structures. Characters were described as follows:

Continuous characters. (1) Total body length (TL) (Figure 2a), (2) pronotal length (PL) (Figure 2e), (3) pronotal width in anterior region (PWA) (Figure 2e), (4) pronotal width in posterior region, (PWP) (Figure 2e), (5) elytral length (EL) (Figure 2e), (6) length of head-pronotum (LHP) (Figure 2a), (7) eye length (EYL) (Figure 2j), (8) height of ocular canthus (HOC) (Figure 2j), (9) width of ocular canthus (WOC) (Figure 2j), (10) eye width (EW) (Figure 2j), (11) mandible length (ML) (Figure 2j), (12) eye height at lateral side (EH) (Figure 2a), (13) head length (HL) (Figure 2b), (14) epistomal width (EPW) (Figure 2d), (15) distance between ocular canthus (DOC) (Figure 2d), (16) distance between procoxae (DCI) (Figure 2b), (17) distance between mesocoxae (DCII) (Figure 2b), (18) distance between metacoxae (DCIII) (Figure 2b).

Meristic character. (19) Number of crenulations on the edge of the elytral disc (NCED). Crenulations are cuticular ornamentations raised and flattened, with convex margins and concave bases. The anterior margin of the elytral disc has crenulations along its entire edge. The size of crenulations decreases from the inner zone to the lateral margin [1,10,16] (Figure 2e). (20) number of crenulations on the third elytral striae (NCE3). The elytral disc has crenulations on the third interstriae of both elytra. These crenulations decrease in size towards the posterior zone, disappearing at the elytral apex (Figure 2f). (21) number of crenulations on the second elytral striae (NCE2) (Figure 2f). Elytral disc exhibits crenulations in the first interstriae, which are conspicuous in the anterior zone and decrease in size until they disappear in the elytral apex. (22) number of tubercles on the third elytral interstriae (NTIE3) (Figure 2f). (23) number of tubercles on the first elytral interstriae (NTIE1) (Figure 2f).

Nominal characters. (24) shape of crenulations located on the elytral disc (SCE) (Figure 2e). Elytral crenulations vary in diameter and depth and can be categorized as: (0) Irregular, (1) Circular. (25) Scale density on elytral declivity (SDED) could be (0) sparse, arranged in a single row of scales in the second interstriae, with space between them (Figure 2f) (1) abundant, two or more rows of scales in the second interstriae, with truly little space between them (Figure 2e) [1,10,16].

### 2.4. Statistical Analyses

#### 2.4.1. Analysis of Sexual Dimorphism

Sexual dimorphism in *P. baumanni* has traditionally been described using ordinal and qualitative traits, such as the number of setae and tubercles on the elytral declivity and the presence of a frontal ridge [1], while other continuous, meristic, and nominal traits have been less explored. We evaluated sexual dimorphism for the 25 characters previously described using a multivariate and univariate approach. From the multivariate perspective, we calculated a Gower distance between the specimens with the R package “vegan” [39] and used the distance matrix to perform a Principal Coordinate Analysis with the R package “ape” [40]. To visualize the ordination pattern, we plotted the first two axes, coloured the points according to the sex of the specimens, and added convex hulls to each group with the R package “graphics” [41]. We calculated the convex hulls with the R package “grDevices” [41]. Then, we tested multivariate differences between sexes with a Permutational Multivariate Analysis of Variance (PERMANOVA) with 1000 iterations with the R package “vegan” [39]. For a univariate perspective, we performed an Analysis of Variance (ANOVA) for each continuous variable and generalized linear models for counts and binary characters with Poisson and Binomial distributions, respectively, using the R package “stats” [41]. We tested the normality and homoscedasticity of model residuals with the visual criteria proposed by Crawley [42]. We assumed normality if the points formed a straight line in a quantile-quantile plot (Q-Q plot), and homoscedasticity if the points formed a homogenous cloud in a plot of fitted values against model residuals. For sexually dimorphic variables, we calculated the mean, standard error, and range. For further analyses, we split the dataset into three subsets: (i) all specimens with non-sexually dimorphic variables (Non-Dimorp), (ii) males with sexually dimorphic variables (Mal-Dimorp), and (iii) females with sexually dimorphic variables (Fem-Dimorp). This partition was used to prevent sex-related variation from being confounded with geographic patterns in subsequent analyses.

#### 2.4.2. Analysis of Geographic Patterns in Morphological Traits

##### Comparisons Among Biogeographic Provinces

*Phloeosinus baumanni* is a widely distributed species, ranging from the United States to El Salvador [8], and occurring across multiple biogeographic provinces. Given this broad distribution, we explored whether the evaluated morphological traits showed broad overlap or discrete grouping among sampled biogeographic provinces. Because sample sizes differed strongly among provinces, this analysis was treated as exploratory and was used primarily to visualize broad-scale patterns of morphospace overlap rather than to infer definitive differences among biogeographic provinces. As a first approach to evaluating these patterns, we analyzed the multivariate ordination of populations in relation to the biogeographic provinces proposed by Tania Escalante et al. [28] and Juan J. Morrone et al. [29]. We calculated a Gower distance per subset (Non-Dimorp, Mal-Dimorp, and Fem-Dimorp) with the R package “vegan” [39] and used each distance matrix to perform a Principal Coordinate Analysis per subset with the R package “ape” [40]. We plotted the first two axes, coloured the points by biogeographic province, and added a convex hull per group, with the R package “graphics” [41]. We calculated each convex hull with the R package “grDevices” [41].

##### Analysis of Differences Among Localities Within the Transmexican Volcanic Belt

To test geographic differences in beetle morphology within a biogeographic province, we selected specimens from the Transmexican Volcanic Belt and included only localities represented by at least 14 specimens. We used a multivariate and univariate approach by selecting only the specimens distributed on the Transmexican Volcanic Belt in each subset (Non-Dimorp, Mal-Dimorp, Fem-Dimorp) and the localities with at least 14 specimens (I to IX, Table 1). For the multivariate perspective, we calculated a Gower distance for each subset with the R package “vegan” [39] and used it to perform a Principal Coordinate Analysis per subset with the R package “ape” [40]. Then, we calculated the group meaning for each locality, plotted it in the first two axes, and selected a point shape per locality. We evaluated multivariate differences per subset among localities by performing a Permutational Multivariate Analysis of Variance (PERMANOVA) with 1000 iterations with the R package “vegan” [39]. For the univariate perspective, we performed an Analysis of Variance (ANOVA) for each continuous variable and generalized linear models for counts with Poisson and Binomial distributions, respectively, using the R package “stats” [41]. We tested the normality and homoscedasticity of model residuals with the visual criteria proposed by Crawley [42]. We assumed normality if the points formed a straight line in a quantile-quantile plot (Q-Q plot), and homoscedasticity if the points formed a homogenous cloud in a plot of fitted values against model residuals. To visualize univariate differences among localities, we selected the four variables that contribute most to the first two principal coordinate axes with a biplot for each dataset (Supplementary Figure S1), generated with the R package “stats” [41]. We obtained the group means and standard error for each locality from the models with the R package “multcomp” [43] and plotted both parameters in error plots with the R package “inecolr” [44].

## 3. Results

### 3.1. Sexual Dimorphism

Specimens were clearly grouped by sex in the principal coordinate analysis, with the first two axes explaining 59.39% of the total variance (Figure 3). This pattern was congruent with significant multivariate differences between sexes detected by PERMANOVA (df = 1, F = 113.54, *p* < 0.001). Of the 25 analyzed variables, 13 exhibited significant sexual dimorphism (Table 2). Males had higher measurements for the following traits: width of ocular canthus (HOC), epistomal width (EPW), and distance between mesocoxae (DCII); whereas females for: total body length (TL), eye length (EYL), head length (HL), distance between ocular canthus (DOC), and distance between procoxae (DCI) (Table 2). In terms of the number of crenulations on elytral interstriae, females had more in the second and third interstriae (NCE2, NCE3; Table 1). Regarding the number of tubercles on elytral striae, males had more in the third elytral striae (NTIE3), and females had more in the first elytral striae (NTIE1; Table 2). Finally, males had scarce scales on the elytral declivity and females had abundant scales (SDED; Table 2). Whereas, the non-sexually dimorphic traits were: pronotal length (PL), pronotal width in anterior region (PWA), pronotal width in posterior region (PWP), elytral length (EL), head–pronotum length (LHP), width of ocular canthus (WOC), eye width (EW), mandible length (ML), eye height at lateral side (EH), distance between metacoxae (DCIII), number of crenulations on the edge of the elytral disc (NCED), and shape of crenulations on the elytral disc (SCE) (Table 2).

### 3.2. Geographic Patterns for Morphological Traits

#### 3.2.1. Biogeographic Provinces

The first two principal coordinate axes explained more than 50% of the total variance in each dataset: 58.13% for non-sexually dimorphic traits (Figure 4a), 51.09% for male dimorphic traits (Figure 4b), and 53.58% for female dimorphic traits (Figure 4c). Across datasets, specimens from the Transmexican Volcanic Belt occupied the broadest region of morphospace and overlapped with specimens from the other sampled biogeographic provinces. Because this province was represented by the largest number of specimens, this broader morphospace should be interpreted as the best-sampled portion of the observed phenotypic range rather than as definitive evidence of greater intrinsic variability.

#### 3.2.2. Differences Among Localities Within the Transmexican Volcanic Belt

Significant morphological differences among localities within the Transmexican Volcanic Belt were detected for non-sexually dimorphic traits (PERMANOVA: df = 8, F-value = 6.98, *p*-value < 0.001), as well as for sexually dimorphic traits in males (PERMANOVA: df = 5, F-value = 5.543, *p*-value < 0.001) and females (PERMANOVA: df = 5, F-value = 4.234, *p*-value < 0.001). Ordination analyses showed that the first two principal coordinate axes explained more than 50% of the variance in all datasets (Figure 5). Specimens from Patzcuaro (VIII) were the furthest away from the other localities when non-sexually dimorphic traits and sexually dimorphic traits of females were analyzed (Figure 5a,c). Conversely, specimens from Iztapalapa (V) were the furthest away when only the sexually dimorphic traits of males were analyzed (Figure 5b). From the univariate perspective, the number of crenulations on the edge of the elytral disc was the only non-sexually dimorphic variable that did not differ significantly among localities (Table S1). For sexually dimorphic traits, five variables in females and four in males did not differ significantly among localities, including NTIE3 and NTIE1, which were comparatively homogeneous in both subsets (Table S2).

Additionally, the set of variables that most contribute to the principal coordinate analyses and discrimination among localities changed among datasets (Supplementary Material, Figure S1). For non-sexually dimorphic traits, the variables were pronotal length (PL: df = 8, F-value = 3.70, *p*-value < 0.001; Table S1), pronotal width in the anterior region (PWA: df = 8, F-value = 3.14, *p*-value = 0.002; Table S1), length of head-pronotum (LHP: df = 8, F-value = 4.04, *p*-value < 0.001; Table S1), and eye width (EW: df = 8, F-value = 13.40, *p*-value < 0.001; Table S1). Specimens from Miguel Hidalgo (VI) had the longest pronotum (Figure 6a), specimens from Huixquilucan (IV) and Texcoco (IX) had the narrowest pronotum in the anterior region (Figure 6b), specimens from Miguel Hidalgo (VI) had the longest head-pronotum (Figure 6c), and specimens from Texcoco (IX) had the narrowest eyes (Figure 6d). The remaining localities formed a group with slight variation (Figure 6).

For sexually dimorphic traits in females, the variables were head length (HL: df = 5, F-value = 2.753, *p*-value = 0.023; Table S2), total length (TL: df = 5, F-value = 2.330, *p*-value = 0.048; Table S2), number of crenulations on the third elytral striae (NCE3: df = 5, F-value = 15.449, *p*-value = 0.009; Table S2), and number of crenulations on the second elytral striae (NCE2: df = 5, F-value = 27.051, *p*-value < 0.001; Table S2). Specimens from Huixquilucan (IV) had the shortest head (Figure 8a), specimens from Miguel Hidalgo (VI) had the longest body (Figure 8b), specimens from Patzcuaro (VIII) had more crenulation on the third (Figure 8c) and the second elytral striae (Figure 8d). The remaining localities were more homogeneous (Figure 8).

Morphological analyses revealed pronounced sexual dimorphism in *P. baumanni*, as evidenced by the clear separation of specimens by sex in multivariate space and by univariate significant differences in over half of the evaluated characters. This pattern is consistent with previous observations in Scolytinae, where dimorphism frequently involves head structures and elements of the elytral declivity [1,3,45,46]. In *P. baumanni*, females exhibited larger body size and more developed head-related traits, whereas males showed greater development of epistomal and intercoxal dimensions. The larger size of females is consistent with patterns observed in bark beetles, where the pioneering sex may benefit from increased size during host colonization [47,48,49,50,51]. Sexual dimorphism also extended to characters of the elytral declivity, with females exhibiting more crenulations and greater scale density, while males showed more developed tubercles. Although these traits are commonly used to discriminate between sexes in scolytine, their functional significance in *P. baumanni* remains unclear and should be evaluated in future behavioural or ecological studies [1,3,52].

Sexual dimorphism in *P. baumanni* has traditionally been recognized through qualitative characters, such as the pattern of tubercles on the elytral declivity, the presence of a frontal carina in females, and frontal tubercles in males [10]. However, these features may be difficult to interpret consistently across specimens and are not always easily visualized in other *Phloeosinus* species [17,53]. Therefore, they were not evaluated here; instead, we provide a quantitative assessment of sexual dimorphism using traits that had not previously been analyzed in this intraspecific context.

## 4. Discussion

Beyond sexual differentiation, part of the morphological variation detected in *P. baumanni* was associated with locality. Phenotype results from the interaction between genotype and environment [54,55,56], both of which may vary within biogeographic provinces, particularly in montane systems such as those inhabited by *P. baumanni*. This is exemplified by the Transmexican Volcanic Belt, a region with a complex climatic and geological history that encompasses much of the observed phenotypic variability [57,58,59]. Specimens from this province occupied a wide morphospace overlapping with other regions, suggesting that morphological variation has a spatial component but is expressed through variable or labile traits rather than clear morphological discontinuities. The absence of consistent clustering among localities and the presence of only partial differentiation suggest that variation reflects a mosaic of conserved and labile traits rather than discrete geographic partitioning. In other scolytine genera, local environmental conditions and host availability may contribute to this pattern [25,48,60,61,62,63,64,65,66]; however, these factors were not directly evaluated in the present study and should therefore be considered as hypotheses for future research. Overall, these patterns are consistent with locality-associated phenotypic variation among specimens currently assigned to *P. baumanni*, as supported by the extensive overlap in morphospace and the lack of clear morphological boundaries among populations. However, morphological evidence alone is insufficient to rule out the possibility of differentiated lineages, and additional molecular or phylogeographic data would be necessary to evaluate this hypothesis more rigorously [67,68].

Regarding the patterns of individual traits, those associated with head morphology and the elytral declivity contributed most strongly to geographic differentiation. These results suggest that sexual dimorphism contributes not only to overall phenotypic variability but also to how morphological differences among localities are expressed [21,69]. These results are consistent with patterns reported in other *Phloeosinus* species, in which morphological variation occurs among populations without clear taxonomic discontinuities [17]. The high phenotypic variability observed in *P. baumanni* is congruent with patterns documented in other bark beetles, where interpopulation variation in certain characters may approach or exceed interspecific differences [25,48,70].

A key implication of these findings concerns the stability and diagnostic value of morphological characters. Several traits that showed significant variation in this study, including body size, cephalic proportions, and elements of elytral ornamentation, have historically been used for species delimitation in Scolytinae [1,25]. However, their sensitivity to both sexual dimorphism and locality-associated variation suggests that they should not be used as standalone indicators of species boundaries, particularly when morphological variability is underestimated due to limited geographic sampling [71,72]. In contrast, more general structural features may exhibit greater evolutionary stability, whereas finer details of surface sculpture and ornamentation are more labile, as has been observed in both extant and fossil Curculionoidea [58,59]. In *P. baumanni*, this distinction is supported by the variability observed in traits associated with cephalic proportions and elytral sculpture.

Some traits, including the height of the ocular canthus (HOC), head length (HL), epistomal width (EPW), and the number of crenulations on the second and third elytral striae (NCE2 and NCE3, see Table S1), showed both sexual dimorphism and locality-associated variation, indicating high intraspecific variability. As such, these traits should be treated with caution in taxonomic contexts, as they may fall within the range of intraspecific variability rather than representing reliable species-level differences. These findings also underscore a broader issue in Scolytine taxonomy: the extent to which intraspecific morphological variability may overlap with characters traditionally used for species delimitation. Previous studies have noted that several diagnostic traits in bark beetles, particularly those related to body size, cephalic proportions, and elytral ornamentation, may vary within species across geographic populations [1,25,73,74]. As a result, interpopulation variation can sometimes approach or even exceed differences observed between closely related species. In this context, documenting patterns of phenotypic variability across widely distributed species becomes particularly important for evaluating the reliability of diagnostic characters and for avoiding overestimating species diversity [75,76]. Our findings support the importance of considering geographic and sexual variation when interpreting morphological differences within the genus *Phloeosinus*.

Taken together, these patterns suggest that the morphometric variation observed in *P. baumanni* may reflect phenotypic responses associated with local conditions during host colonization, rather than fixed morphological discontinuities. In bark beetles, host quality, colonized tissue, larval development, and environmental conditions can influence adult size and external structures; therefore, differences in cephalic proportions and elytral sculpture may have biological meaning within the species [17,21,25,69,70]. Nevertheless, because these factors were not directly tested and no molecular data were included, the results should be interpreted strictly as phenotypic evidence and as a basis for future integrative evaluation [58,59,71,72].

The comparison with morphologically similar species such as *P. variolatus* is particularly relevant because it emphasizes that some diagnostic traits traditionally used in *Phloeosinus*, especially those related to elytral sculpture, declivity ornamentation, and vestiture, may show overlapping variation. A closely related example that illustrates this issue is the distinction between *P. baumanni* and *P. variolatus*, which have traditionally been separated based on subtle differences in body proportions, pronotal sculpture, and elytral characters [1]. Notably, many of these same traits were found here to vary significantly within *P. baumanni*, raising the possibility that some diagnostic features used at the intrageneric level may fall within the range of intraspecific variation. Thus, the observed variability in *P. baumanni* does not necessarily indicate the presence of discrete taxonomic entities but rather highlights the need to evaluate diagnostic characters within a broader intraspecific framework before assigning taxonomic significance to subtle phenotypic differences. Although certain structural characters, such as the development of the second interstria on the elytral declivity, have been proposed as more consistent, their stability across populations remains to be critically evaluated.

## 5. Conclusions

Overall, the results indicate that phenotypic variation in specimens assigned to *P. baumanni* is associated with both sexual dimorphism and locality-level differences [9,63]. Some characters traditionally used in the taxonomy of *Phloeosinus*, particularly those related to body size, cephalic proportions, and elytral ornamentation, exhibited intraspecific variability, highlighting the importance of broad geographic sampling when evaluating their diagnostic value. Although the absence of clear morphological discontinuities does not support recognizing discrete groups based solely on the characters analyzed here, our conclusions should be interpreted strictly within a phenotypic framework and should not be considered formal evidence for or against lineage differentiation. Instead, the detected patterns provide a useful framework to guide future, more targeted sampling efforts. In particular, localities or populations showing greater phenotypic variability could be prioritized in subsequent integrative studies, as they may represent key areas for evaluating population-level variation, the stability of diagnostic characters, and the potential presence of cryptic diversity. Therefore, the integration of molecular, phylogeographic, and ecological data will be essential to better understand the processes underlying the observed patterns of variation.

## Figures and Tables

**Table 1:** Location, coordinates, and host identity of the 21 *P. baumanni* collection sites studied. Abbreviations: I, Amecameca; II, Coyoacán; III, Cuauhtémoc; IV, Huixquilucan; V, Iztapalapa; VI, Miguel Hidalgo; VII, Naucalpan; VIII, Pátzcuaro (Michoacán); IX, Texcoco; X, localities in Guerrero; XI, localities in the Chiapas Highlands; XII, remaining locality in Michoacán; XIII, remaining localities in the eastern Transmexican Volcanic Belt; XIV, locality in Nuevo León; TVBe, Transmexican Volcanic Belt (eastern sector); TVBw, Transmexican Volcanic Belt (western sector); CHIS, Chiapas Highlands; SMOR, Sierra Madre Oriental; SMS, Sierra Madre del Sur; BP, Biogeographic province.

ID	BP	State, Municipality and Locality			Number of Specimens (Collection)	Latitude	Longitude	Host
I	TVBe	Edo.Mx	Amecameca	-	6 (CNIN)	19°07′42.29″	98°46′7.88″	*Hesperocyparis* sp.
I	TVBe	Puebla	Amecameca	Iztaccíhuatl	14 (CNIN)	19°10′21.78″	98°38′27.80″	*Hesperocyparis* sp.
II	TVBe	CDMX	Coyoacán	C.U.	59 (CNIN)	19°18′35.88″	99°10′38.29″	*H. lusitanica*
III	TVBe	CDMX	Cuauhtémoc	Alameda central	23 (CNIN)	19º43′44”	99º14′4”	*Hesperocyparis* sp.
IV	TVBe	Edo.Mx	Huixquilucan	La Herradura	26 (LARSF-SEMARNAT)	19°21′43.79″	99°20′47.40″	*Hesperocyparis* sp.
V	TVBe	CDMX	Iztapalapa	Iztapalapa de Coas	16 (LARSF-SEMARNAT)	19°20′31.50″	99°03′11.58″	*Hesperocyparis* sp.
VI	TVBe	CDMX	Miguel Hidalgo	Campo Militar	20 (LARSF-SEMARNAT)	19°25′28.54″	99°11′50.34″	*H. lusitanica*
VII	TVBe	Edo.Mx	Naucalpan	Parque Naucalli	28 (UACH)	19°29′37.80″	99°14′36.57″	*Hesperocyparis* sp.
VIII	TVBw	Michoacán	Pátzcuaro	Esc. Sec. No. 3	30 (IPN)	19°31′21.29″	101°36′36.6″	*H. lusitanica*
IX	TVBe	Edo.Mx	Texcoco	Sn Luis Huexotla	14 (UACM)	19°28′50.33″	98°51′54.81″	*Hesperocyparis* sp.
X	SMS	Guerrero	Acapulco	El Marqués	2 (IPN)	16°47′42.35″	99°49′11.78″	*H. lusitanica*
X	SMS	Guerrero	Leonardo Bravo	Ejido Yextla	5 (IPN)	17°39′33.39″	99°40′17.25″	*Hesperocyparis* sp.
XI	CHIS	Guatemala	Huehuetenango	Huehuetenango	2 (CNIN)	15°19′16.35″	91°28′12.96″	*H. lusitanica*
XI	CHIS	Guatemala	Sacatepéquez	La Cumbre	1 (CNIN)	14°35′43.05″	90°41′9.69″	*H. lusitanica*
XII	TVBw	Michoacán	Morelia	Bosque Cuauhtémoc	3 (ENCB)	19°41′48.4″	101°10′53″	*H. lusitanica*
XIII	TVBe	CDMX	Mag. Contreras	C. Pedregal Sn Ángel	5 (INIFAP)	19°19′59.72″	99°12′47.88″	*H. lusitanica*
XIII	TVBe	Edo.Mx	Metepec	Rancho Guadalupe	1 (INIFAP)	19°14′46.61″	99°34′47.18″	*Hesperocyparis* sp.
XIII	TVBe	Edo.Mx	Nicolas Romero	Granjas Esclavo	2 (INIFAP)	19°36′52.28″	99°17′43.4″	*Hesperocyparis* sp.
XIII	TVBe	Morelos	Cuernavaca	Cuernavaca	6 (UACH)	18°55′7.29″	99°13′44.57″	*Hesperocyparis* sp.
XIII	TVBe	Morelos	Yecapixtla	Xochitlán	1 (UACH)	18°52′50.95″	98°49′07.33″	*H. lusitanica*
XIV	SMOR	Nuevo León	Iturbide	-	4 (CNIN)	24°42′33.22″	99°54′18.33″	*H. arizonica*

**Table 2:** Differences between sexes on morphological traits of *Phloeosinus baumanni*. Results of analysis of variance for continuous variables and generalized linear models for counts (Poisson distribution) and nominal variables (binomial distribution). Summary of measurements of central tendency (mean) and dispersion (standard error and range) for continuous variables and counts. For nominal variables, the frequency of each character state per sex. Variables with significant sexual dimorphism are in bold. Statistics for continuous variables were F-value, whereas for counts and nominal variables were Chisq. Abbreviations: ANOVA, analysis of variance; DCI, distance between procoxae; DCII, distance between mesocoxae; DCIII, distance between metacoxae; df, degrees of freedom; DOC, distance between ocular canthus; EH, eye height at lateral side; EL, elytral length; EPW, epistomal width; EW, eye width; EYL, eye length; glm, generalized linear models; HL, head length; HOC, height of ocular canthus; LHP, head–pronotum length; ML, mandible length; NCE2, number of crenulations on the second elytral striae; NCE3, number of crenulations on the third elytral stria; NCED, number of crenulations on the edge of the elytral disc; NTIE1, number of tubercles on the first elytral interstria; NTIE3, number of tubercles on the third elytral interstria; PL, pronotal length; PWA, pronotal width in anterior region; PWP, pronotal width in posterior region; SCE, shape of crenulations on the elytral disc; SDED, scale density on the elytral declivity; TL, total body length; WOC, width of ocular canthus.

Type of Variable	Dependent Variable	ANOVA/glm			Summary per Sex	
		df	Statistic	*p*-Value	Males	Females
Continuous	**TL**	1	7.021	0.008	3785.18 ± 26.99 (3033.28–4701.08)	3890.92 ± 29.37 (3139.38–4913.15)
	PL	1	1.01	0.316	1466.13 ± 10.35 (1196.10–1790.05)	1450.89 ± 11.07 (1091.64–1825.53)
	PWA	1	2.028	0.156	1747.02 ± 11.74 (1426.22–2133.24)	1772.30 ± 13.30 (1441.56–2266.76)
	PWP	1	2.572	0.110	658.37 ± 9.11 (412.17–963.20)	678.31 ± 8.46 (473.42–923.20)
	EL	1	2.108	0.148	2327.67 ± 18.57 (1856.25–2855.73)	2363.80 ± 18.54 (1915.85–3057.58)
	LHP	1	0.053	0.819	1622.13 ± 12.61 (1295.06–1961.90)	1626.26 ± 12.79 (1339.17–2039.31)
	**EYL**	1	17.408	<0.001	620.17 ± 6.27 (355.39–774.32)	658.17 ± 6.37 (453.65–843.17)
	**HOC**	1	14.701	<0.001	272.99 ± 3.27 (133.31–336.04)	255.87 ± 3.04 (170.03–362.91)
	WOC	1	0.975	0.324	115.68 ± 2.12 (59.62–205.48)	112.85 ± 1.91 (52.17–193.86)
	EW	1	0.033	0.857	182.44 ± 2.16 (101.35–237.46)	183.04 ± 2.54 (113.21–251.78)
	ML	1	1.848	0.175	276.56 ± 5.31 (149.42–405.36)	287.43 ± 5.97 (98.68–448.56)
	EH	1	1.418	0.235	861.73 ± 8.25 (652.04–1230.38)	847.92 ± 8.14 (600.24–1054.60)
	**HL**	1	39.959	<0.001	1083.72 ± 9.34 (801.99–1350.35)	1169.61 ± 9.86 (912.88–1455.29)
	**EPW**	1	18.438	<0.001	653.50 ± 6.18 (433.53–755.36)	614.63 ± 6.61 (433.78–813.27)
	**DOC**	1	72.975	<0.001	869.91 ± 7.25 (590.91–1078.46)	956.22 ± 7.03 (638.92–1149.24)
	**DCI**	1	5.708	0.018	293.66 ± 5.34 (146.73–478.03)	311.60 ± 5.28 (168.07–521.86)
	**DCII**	1	9.826	0.002	487.64 ± 9.00 (214.18–712.92)	446.36 ± 9.60 (249.13–830.52)
	DCIII	1	3.767	0.053	472.40 ± 7.69 (234.58–679.57)	492.4 ± 6.55 (206.11–641.25)
Counts	NCED	1	0.848	0.357	12.14 ±0.08 (10–15)	12.54 ± 0.08 (11 –15)
	**NCE3**	1	74.770	<0.001	12.59 ± 0.28 (5–20)	16.64 ± 0.23 (10–23)
	**NCE2**	1	75.918	<0.001	7.53 ± 0.27 (2–17)	10.76 ± 0.28 (5–18)
	**NTIE3**	1	7.473	0.006	8.65 ± 0.09 (6–11)	7.69 ± 0.08 (5–10)
	**NTIE1**	1	590.36	<0.001	1.30 ± 0.05 (0–3)	7.16 ± 0.09 (5–9)
Nominal	SCE	1	0.987	0.325	0 (0.316); 1(0.684)	0 (0.370); 1(0.630)
	**SDED**	1	343.830	<0.001	0 (0.992); 1(0.008)	0 (0.008); 1 (0.992)

## Data Availability

The original contributions presented in this study are included in the article and Supplementary Materials. Further inquiries can be directed to the corresponding authors.

## Supplementary Materials

The following supporting information can be downloaded at: https://www.mdpi.com/article/10.3390/insects17060625/s1.
