# Supplementary material for: The Phenotypic Landscape of Phloeosinus baumanni: Spatial Patterns of Morphological Variation and Their Implications for Ecology and Taxonomy
Source: Insects. 2026 Jun 14;17(6):625. doi: 10.3390/insects17060625 (PMC13300303; doi:10.3390/insects17060625)
Supplement: Supplementary file 1 [file insects-17-00625-s001.zip › insects-4357834-supplementary.pdf]

Table S1. Differences among localities on non-sexually dimorphic traits of *Phloeosinus baumanni.* Results of analysis of variance for continuous variables and generalized linear models for counts (Poisson distribution). Geographically structured variables are shown in bold. Statistics for continuous variables were F-value, whereas for count variables were Chisq. Abbreviations: PL, pronotal length; PWA, pronotal width in anterior region; PWP, pronotal width in posterior region; EL, elytral length; LHP, head–pronotum length; WOC, width of ocular canthus; EW, eye width; ML, mandible length; EH, eye height at lateral side; DCIII, distance between metacoxae; NCED, number of crenulations on the edge of the elytral disc; df, degrees of freedom.

Type of variable	Dependent variable	df	Statistic	p-value
Continuous	**PL**	**8**	**3.697**	**<0.001**
	**PWA**	**8**	**3.136**	**0.002**
	**PWP**	**8**	**11.424**	**<0.001**
	**EL**	**8**	**3.765**	**<0.001**
	**LHP**	**8**	**4.037**	**<0.001**
	**WOC**	**8**	**11.491**	**<0.001**
	**EW**	**8**	**13.399**	**<0.001**
	**ML**	**8**	**2.904**	**0.004**
	**EH**	**8**	**6.380**	**<0.001**
	**DCIII**	**8**	**9.939**	**<0.001**
Counts	NCED	8	3.552	0.895

Table S2. Differences among localities on sexually dimorphic traits of *Phloeosinus baumanni.* Results of analysis of variance for continuous variables and generalized linear models for counts (Poisson distribution). Geographically structured variables are shown in bold. Statistics for continuous variables were F-value, whereas for count variables were Chisq. Abbreviations: TL, total body length; EYL, eye length; HOC, height of ocular canthus; HL, head length; EPW, epistomal width; DOC, distance between ocular canthus; DCI, distance between procoxae; DCII, distance between mesocoxae; NCE3, number of crenulations on the third elytral striae; NCE2, number of crenulations on the second elytral striae; NTIE3, number of tubercles on the third elytral interstriae; NTIE1, number of tubercles on the first elytral interstriae; df, degrees of freedom.

Type of variable	Dependent variable	Dataset	df	Statistic	p-value
Continuous	TL	Male	5	1.832	0.114
		**Female**	**5**	**2.330**	**0.048**
	EYL	Male	5	1.374	0.242
		**Female**	**5**	**4.228**	**0.002**
	HOC	**Male**	**5**	**6.738**	**<0.001**
		**Female**	**5**	**2.504**	**0.036**
	HL	**Male**	**5**	**6.468**	**<0.001**
		**Female**	**5**	**2.753**	**0.023**
	EPW	**Male**	**5**	**4.602**	**<0.001**
		**Female**	**5**	**4.473**	**0.001**
	DOC	**Male**	**5**	**4.789**	**<0.001**
		Female	5	0.567	0.725
	DCI	**Male**	**5**	**2.515**	**0.035**
		Female	5	1.680	0.147
	DCII	**Male**	**5**	**7.746**	**<0.001**
		Female	5	2.043	0.079
Counts	NCE3	**Male**	**5**	**15.098**	**0.010**
		**Female**	**5**	**15.449**	**0.009**
	NCE2	**Male**	**5**	**43.753**	**<0.001**
		**Female**	**5**	**27.051**	**<0.001**
	NTIE3	Male	5	0.910	0.970
		Female	5	1.462	0.917
	NTIE1	Male	5	2.373	0.796
		Female	5	4.413	0.492

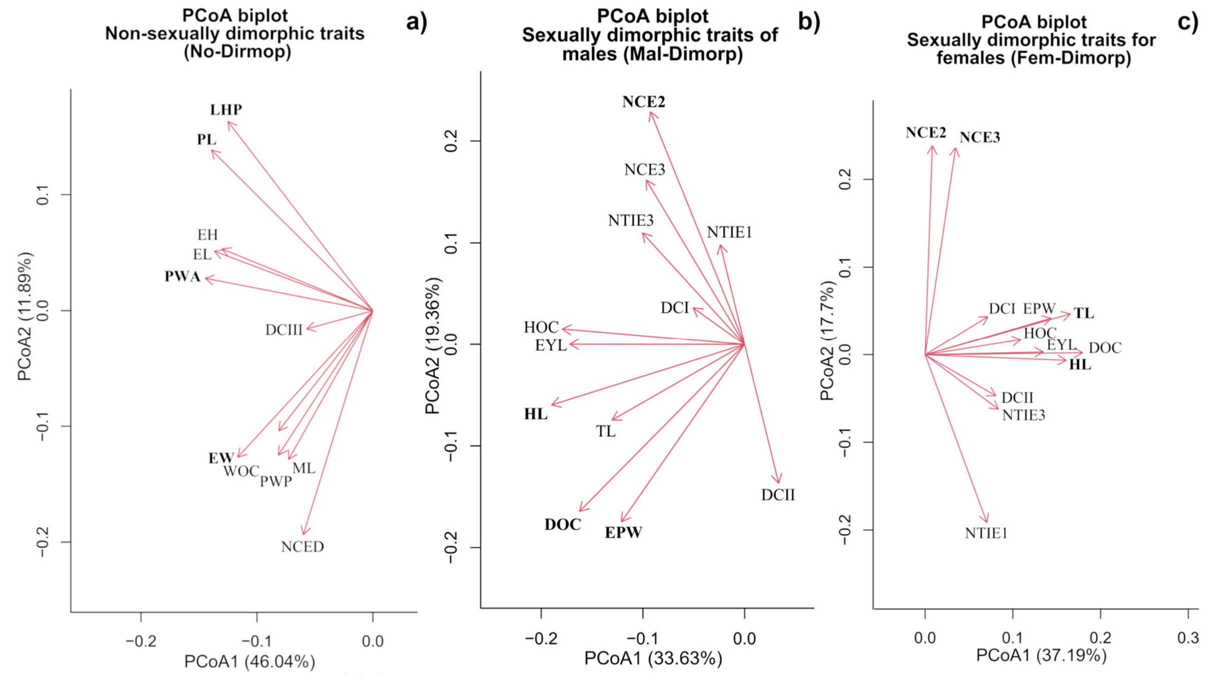

Fig S1. Contribution of variables to Principal Coordinate Analyses for three datasets of variables. a) Non-sexually dimorphic traits. b) Sexually dimorphic traits in males. c) Sexually dimorphic traits in males. Bold abbreviations indicate variables significantly different among localities within the Transmexican Volcanic Belt and contribute more to the Principal Coordinate Axes. Abbreviations: TL, total body length; PL, pronotal length; PWA, pronotal width in anterior region; PWP, pronotal width in posterior region; EL, elytral length; LHP, head–pronotum length; EYL, eye length; HOC, height of ocular canthus; WOC, width of ocular canthus; EW, eye width; ML, mandible length; EH, eye height at lateral side; HL, head length; EPW, epistomal width; DOC, distance between ocular canthus; DCI, distance between procoxae; DCII, distance between mesocoxae; DCIII, distance between metacoxae; NCED, number of crenulations on the edge of the elytral disc; NCE3, number of crenulations on the third elytral stria; NCE2, number of crenulations on the second elytral stria; NTIE3, number of tubercles on the third elytral interstria; NTIE1, number of tubercles on the first elytral interstria.
